# Design-of-Experiments (DoE)-Assisted Fabrication of Quercetin-Loaded Nanoemulgel and Its Evaluation against Human Skin Cancer Cell Lines

**DOI:** 10.3390/pharmaceutics14112517

**Published:** 2022-11-19

**Authors:** Aman Chitkara, Bharti Mangla, Pankaj Kumar, Shamama Javed, Waquar Ahsan, Harvinder Popli

**Affiliations:** 1 Department of Pharmaceutics, Delhi Pharmaceutical Sciences and Research University, New Delhi 110017, India; 2 Department of Pharmaceutics, College of Pharmacy, Jazan University, P.O. Box 114, Jazan 82817, Saudi Arabia; 3 Department of Pharmaceutical Chemistry and Pharmacognosy, College of Pharmacy, Jazan University, P.O. Box 114, Jazan 82817, Saudi Arabia

**Keywords:** quercetin (QCT), nanoemulgel, skin cancer, nanoemulsion, bioavailability

## Abstract

**Background:** Quercetin (QCT) is a natural polyphenolic flavonoid showing great potential in the treatment of skin cancer. However, its use is limited owing to its poor water solubility, poor absorption, quick metabolism and excretion, as well as low stability. Preparation of nanoemulgel has been proven to be an effective approach to deliver the drugs topically due to various advantages associated with it. **Objectives:** This study aimed to prepare stable nanoemulgel of QCT using a Design-of-Experiments (DoE) tool for optimization, to characterize and to assess its in vivo toxicity and efficacy against human cancer cell lines in vitro. **Methods:** An ultrasonication emulsification method was used for the preparation of QCT-loaded nanoemulsion (QCT@NE). Box–Behnken design was used for the optimization of developed nanoemulgel. Then, in vitro characterization of prepared nanoemulsion was performed using Fourier Transform-Infra Red (FT-IR) spectroscopy, Scanning Electron Microscopy (SEM), particle size analysis, determination of zeta potential and entrapment efficiency (%EE). Further, the developed QCT-loaded nanoemulgel (QCT@NG) was characterized in vitro using texture profile analysis, viscosity and pH determination. Eventually, the cell cytotoxicity studies of the prepared nanoemulgel were performed on the skin cancer cell lines A431 followed by an acute toxicity and skin irritation study on male wistar rats in vivo. **Results:** The developed QCT@NE was found to be nanometric in size (173.1 ± 1.2 nm) with low polydispersity index (0.353 ± 0.13), zeta potential (−36.1 ± 5.9 mV), and showed good %EE (90.26%). The QCT@NG was found to be substantially more effective against the human skin carcinoma (A431) cell lines as compared to plain QCT with IC_50_ values of 108.5 and 579.0 µM, respectively. Skin irritation study showed no sign of toxicity and ensured safety for topical application. Hematological analysis revealed no significant differences between the treatment and control group in any biochemical parameter. In the nanoemulgel treatment group, there were no discernible differences in the liver enzymes, bilirubin, hemoglobin, total leukocyte and platelet counts as compared to the control group. **Conclusions:** The optimized QCT@NG was found to be an ideal and promising formulation for the treatment of skin cancer without showing skin irritation and organ toxicity.

## 1. Introduction

One of the most prevalent forms of cancer characterized by malignant growth is skin cancer, which can result in skin deformity [1]. According to the reports from the Indian Council of Medical Research (ICMR), the incidence of skin cancer in the northern and northeast regions of India is higher than other parts, with 1.62 cases per 100,000 men and 1.21 cases per 100,000 women [2]. Family history, a prior melanoma occurrence, gene polymorphisms, multiple moles, UV sensitivity, immunological suppression, alcohol use, and ultraviolet radiation exposure are only a few of the risk factors for skin cancer [3]. Based on the type of cell from which it arises, skin cancer is categorized into four groups, namely melanoma, squamous cell carcinoma (SCC), basal cell carcinoma (BCC), and Merkel cell carcinoma [4]. According to several studies, sunburn and UV-B sunlight exposure make people more prone to developing skin cancer. Sun exposure also mutates the p53 gene, which is crucial for the initiation and progression of skin cancer. The p53 protein participates in cell death by assisting DNA repair or eliminating cells with severe DNA damage, acting as a “custodian of the genome” [5]. Skin cancer may currently be treated with a number of different methods, including excisional surgery, Moh’s surgery, radiation therapy, chemotherapy, photodynamic therapy, etc. However, because these treatments are artificial, they come with a number of negative effects, including pain, swelling, lymphedema, bleeding, and nerve damage. Nowadays, herbal medications containing phytonutrients have shown promising potential in the treatment of skin cancer, diabetes, cardiovascular diseases, etc. [6]. 

In recent years, natural bioactive compounds, including flavonoids, have garnered considerable interest owing to their vast array of biological activities, such as anti-inflammatory, antioxidant, and anti-proliferative activities. Quercetin (QCT) is one such naturally occurring polyphenolic flavonoid that is chemically represented as 3,3’,4’,5,7 pentahydroxy flavone (C_15_H_10_O_7_). It is frequently found in numerous food plants, including capers, lovage, dill, cilantro, onions, and apple and is one of the most effective antioxidant that inhibits pro-inflammatory cytokines such as IL-6, chemokines, TNF-, nitric oxide, IL-1, etc. [7]. It is reported to have antiallergic, anti-inflammatory, antioxidant, anti-genetic, and antiviral properties, which can be helpful in the prevention of cancer [8]. Phase II metabolic enzymes found in the epithelial cells of the stomach and intestines metabolize QCT, which is readily absorbed in the digestive system. It undergoes further processing in the kidney and liver, resulting in its first pass metabolism [9]. Despite the great pharmacological significance of QCT, its poor water solubility, poor absorption, quick metabolism, rapid systemic elimination, and low stability restrict its medicinal uses. Owing to its poor water solubility and side effects, oral administration of QCT frequently leads to low patient compliance. A topical formulation is therefore warranted to address these issues which can result in its improved water solubility, facilitated skin permeation, reduced fluctuations in plasma drug concentration, and promotes controlled drug release [10]. In comparison to other methods, the topical route of administration has potential benefits which include avoiding first-pass hepatic metabolism, extending the duration of action, minimizing unwanted side effects, enhancing pharmacological efficacy, and improving patient compliance. Most notably, the topical route of drug administration can effectively be used when long-term or chronic medication therapy is needed.

Nanoemulsions have recently received a lot of attention as potential new carriers for effective topical administration. Nanoemulsion is a liquid formulation that is pharmacokinetically stable and has a droplet diameter between 10 to 100 nanometers. Nanoemulsions have various advantages over conventional formulations, including increased drug solubility, easier penetration to deeper skin layers, decreased dose and therefore dose-related side effects [11]. However, the low viscosity and short retention period of nanoemulsions beset its topical applicability, and instead, nanoemulgels can be prepared from nanoemulsions by adding appropriate gelling agents, such as Carbopol 940 and Carbopol 934. An effective method for using nanotechnology to distribute pharmaceuticals is called a nanoemulsion-based gel system. It is thought to improve the biopharmaceutical properties of drugs that are not easily soluble [12]. Nanoemulgels display improved applicability, thixotropic behavior, greaseless nature, improved spreadability, controlled rheological properties, and combined properties of emulsions and gels. They possess a consistency similar to jelly and offer a wide range of applications as dermatological products [13,14].

Therefore, in this study, QCT@NG was intended to be fabricated using Carbopol 940 as a gelling agent with the help of a Design-of-Experiments (DoE) program using Box–Behnken Design. The prepared nanoemulgel was characterized using spectroscopic and microscopic techniques, followed by evaluation of its anticancer potential and acute toxicity. This study was aimed to develop and optimize QCT@NG to achieve improved efficacy of QCT against skin cancer cells by enhancing its solubility and simultaneously the permeability across the skin barrier. 

## 2. Materials and Methods

### 2.1. Materials

Quercetin (QCT) was obtained as a gift sample from Arjuna Naturala Ltd (Kerala, India), whereas extra-pure olive oil (>99%) and polyethylene glycol 400 were provided by Loba Chemie (Mumbai, India) and Abitec (ABF ingredient company interprise, Bengaluru, India), respectively. Carbopol 940 (98%) was purchased from the Lubrizol Corporation (Wickliffe, OH, USA) and Tween 80 (98.5%) was procured from the Himedia Corporation (Mumbai, India). The MiliQ plus (Millipore, Darmstadt, Germany) system was used to produce the ultra-pure double-distilled water which was used throughout the process. All the other chemicals, reagents and solvents, such as triethylamine, methanol, etc., were of analytical grade and purchased from Sigma Aldrich (Steinhim, Germany).

### 2.2. Methodologies

#### 2.2.1. Preparation of Stock Solution and Calibration Curve 

The standard stock solution of QCT was prepared by dissolving 10 mg of QCT in 100 mL methanol to achieve a concentration of 100 µg/mL. A working standard solution of 10 µg/mL was prepared from the standard stock solution and scanned over the UV range 400–200 nm, and the wavelength of maximum absorbance (λ_max_) was determined. Further, six serial dilutions from the stock solution (2–12 µg/mL) were prepared and their absorbances were measured at the λ_max_. Calibration curve was plotted by keeping concentration on X axis and absorbance on Y axis. 

#### 2.2.2. Solubility Studies

The saturation solubility method was used for the determination of QCT solubility in various oils (olive oil, coconut oil, soy oil, turpentine oil) and surfactants (PEG 400, tween 80, and Transcutol HP) [15]. A measured excess of QCT was added in a glass vial containing oil or surfactant solution (1% *w*/*v*), sealed and stirred continuously at room temperature at 200 rpm for 24 h. The obtained solution was then subjected to centrifugation at 3000 rpm, and supernatant was collected and diluted with methanol. QCT was quantified using the ultraviolet (UV) spectrophotometric method by measuring absorbance at 258 nm. All the experiments were performed in triplicate and the results are expressed as mean ± SD.

#### 2.2.3. Design-of-Experiment (DoE)

Proper optimization is helpful in the prevention of cracking or phase separation of a nanoemulsion. A three-level Box–Behnken experimental design (BBD) was used to optimize the nanoemulsion preparation parameters. The concentration of oil (olive oil, oleic acid), surfactant (PEG 400) and co-surfactants (Tween 80) were considered as independent variables, whereas globule size and entrapment efficiency were regarded as dependent variables. It developed 17 runs with minimum and maximum range of oil (2.5–5 mL), surfactant (4–8 mL) and co-surfactant (4–8 mL). Characterization parameters, such as globule size and drug entrapment efficiency, of nanoemulsion were assessed and optimum parameters were selected on the basis of obtained results.

The Box–Behnken design was used to optimize the formulation parameters using Design-Expert^®^ 7.0.0 software. Parameters such as concentration of oil (mL), surfactant (mL), and co-surfactant (mL) were considered as independent variables. In contrast, mean globule size (Y1), PDI (Y2) and entrapment efficiency (Y3) were considered as dependent variables [15]. Low (−), medium (0), and high (+) values were assigned to the three independent variables of oil concentration (X1), surfactant concentration (X2), and co-surfactant concentration (X3). 

Using the Box–Behnken statistical design tool, the following equation (1) was obtained for the responses:

Y = b_0_ + b_1_ A + b_2_ A + b_3_ C + b_12_ AB + b_13_AC + b_23_ BC + b_11_A^2^ + b_22_B^2^ + b_33_C (1)

where Y is the response related to each factor; b_0_ is the intercept; b_1_, b_2_ and b_3_ are linear coefficients; b_12_, b_13_ and b_23_ are interaction coefficients; and b_11_, b_22_ and b_33_ are quadratic coefficients generated from experimental runs, while A, B and C are independent variables and AB, AC, BC, A^2^, B^2^, C^2^ are quadratic terms, respectively.

#### 2.2.4. Preparation of QCT@NE

The ultrasonication emulsification method was used for the preparation of QCT@NE [16]. Initially, a specific amount of QCT (10 mg) was dissolved in olive oil and oleic acid (7.5 mL) followed by the addition of surfactant (3 mL) and co-surfactant (3 mL). The resultant mixture was stirred properly, dispersed in the oil phase and vortexed for 15 min at 300 rpm. The resultant mixture was then added to the aqueous phase (36.5 mL) slowly, with continuous stirring using a magnetic stirrer for 15 min, and again vortexed for 20 min. Finally, the prepared nanoemulsion was left aside for 24 h to achieve the equilibration. 

#### 2.2.5. Characterization of QCT@NE

##### Determination of Globule Size, Zeta Potential and Polydispersity Index (PDI)

The average globule size, zeta potential and PDI of prepared QCT@NE were measured by the dynamic light scattering (DLS) technique using a Zetasizer Nano-ZS instrument (Malvern, UK). Prior to the analysis, samples were diluted with double-distilled water to make sure that the light scattering intensity was within the sensitivity range of the instrument. All the measurements were made at 25 °C. The PDI values were determined using the same instrument, which indicated the distribution of size range in the tested nanoemulsion, whereas the zeta potential indicated the electrophoretic mobility [17].

##### Entrapment Efficiency (EE)

The UV spectrophotometric method was used to determine the concentration of entrapped QCT in the developed QCT@NE [18]. An aliquot of 2 mL of optimized QCT@NE was centrifuged at 3500 rpm for 30 min, followed by collection of the supernatant liquid. It was diluted using 10 mL of the double-distilled water and the concentration of QCT was determined using UV spectroscopy. The %EE was calculated using the following Equation (2):

(2) $$EE(\%)\;=\;\frac{Ct-Cs}{Ct}\;\times \;100$$

where *Ct* = total drug concentration and *Cs* = concentration of drug in the supernatant.

##### Scanning Electron Microscopy (SEM)

The SEM instrument (Leo 1455 VP, Leo Zeiss, Cambridge, UK) was used to analyze the surface morphology of the prepared QCT@NE. Before imaging, the material was deposited onto aluminium stubs, dried, and then platinum was sputter-coated using an Auto Fine Platinum Coater [19].

##### Attenuated Total Reflectance-Fourier Transform-Infra Red (ATR-FTIR) Analysis 

The FT-IR is one of the most important parameters that provide information regarding the interaction of drugs with their excipients. The FT-IR spectra of pure QCT and QCT@NE were obtained using an ATR-FTIR (Bruker OPTIK GmbH Rudolf-Plank-sir.27 D76275, Ettlingen, Germany) instrument in the range of 4000–400 cm^−1^ [20].

##### In Vitro Drug Release 

An in vitro drug release study was performed using a dialysis membrane (MWCO 12400 Da, Sigma Aldrich, Germany) in the Franz diffusion cell apparatus [21]. An aliquot of 10 mL of the phosphate-buffered saline (PBS) at pH 7.4, mixed with 5% sodium dodecyl sulphate (SDS), was taken in the receptor compartment, whereas 750 μL of the sample was placed in the donor compartment of a diffusion cell followed by continuous stirring for 4 h at 600 rpm. The dialysis membrane was used to separate the donor and receptor compartments. At predetermined intervals (0, 1, 2, 3 and 4 h), samples were taken out and an equal volume of fresh release medium was added to keep the sink condition. The concentration of QCT in the obtained samples was determined using UV-spectroscopy.

#### 2.2.6. Preparation of QCT-loaded Nanoemulgel (QCT@NG)

The QCT@NG was prepared by adding Carbopol 940 gelling agent to the optimized QCT@NE by mixing in a ratio of 1:1, as suggested in the previously reported literature [22]. The mixture was subjected to continuous mixing in order to achieve homogeneity and to form clear nanoemulgel. Triethanolamine (0.1 mL) was added to the prepared QCT@NG in order to maintain the pH. The developed QCT@NG was then characterized using in vitro methods.

#### 2.2.7. Characterization of QCT@NG

##### Measurement of pH

The pH of the developed nanoemulgel was measured using a digital pH meter [23]. Prior to analysis, the pH meter was calibrated using buffer solutions of pH values 4, 7, and 9. All the analyses were made in triplicate and the mean ± SD values were calculated and reported. 

##### Viscosity

Viscosity of optimized QCT@NG was determined using an Anton Paar Rheometer (Anton Paar-Strabe 208054, Graz, Austria) at 25 ± 0.5 °C with spindle number 25 [24]. The sample was placed on the stage and allowed to move downwards for estimating viscosity. The measurements were made in triplicate and the mean ± SD value was calculated.

##### Drug Content 

For the estimation of drug content, 1 g of nanoemulgel was diluted with measured quantity of methanol and the drug content was determined using UV spectroscopy [23].

##### Texture Profile Analysis

The texture profile analysis was performed using a Texture Analyser (XT Plus, Stable Micro Systems Ltd., Surrey, UK) instrument using a reported procedure [25]. Various parameters such as consistency, firmness, cohesiveness, and work of cohesion of nanoemulgel were estimated. A 100 mL glass beaker was filled with 50 mg of the optimized QCT@NG, and a 35 mm diameter disk was placed into it. The probe (A/BE-d35) was set at 5.0 mm/sec with a 10 mm distance during analysis. Utilizing the force–time plot created during analysis, all the parameters were determined. The analysis was performed in triplicate and data were presented as mean ± SD.

#### 2.2.8. Stability Study

The developed nanoemulgel was subjected to stability studies using ICH guidelines [26]. Weighed quantities (500 mg) of the formulation were stored at two different temperatures: low (2–8 °C) and high (40 °C) for a period of two months. After a specific period of time (0, 30 and 60 days), samples were withdrawn and visually examined for physical changes, if any, in the nanoemulgel. Additionally, the drug content and pH were determined to assess the stability using the above-mentioned methods.

#### 2.2.9. Percent Cell Cytotoxicity

% Cell cytotoxic effects were estimated using MTT assay on human skin cancer cell lines (A431) [27]. The cells were seeded in 96 well plates in 100 µL of Dulbecco Modified Eagle’s (DMEM) culture media and then incubated in a humidified chamber at 37 °C with 5% CO_2_ for 24 h. The cells were then treated with plain QCT suspension and QCT@NG. After 72 h, MTT reagent was added into each well plate and incubated for another 2 h. DMSO was added to dissolve the crystals of formazan, and absorbance was measured at 550 nm on a microplate reader and % cell cytotoxicity was calculated.

#### 2.2.10. Animal Studies 

The studies involving animals were approved by the Institutional Animal Ethical Committee of Delhi Pharmaceutical Science and Research University, New Delhi, with registration number 215/GO/ReBi/S/2000/CPCSEA and protocol number IAEC/RCPIPER/2022/I-24. 

##### Skin Irritation Study

Male wistar rats (160–200 g) were used for the skin irritation study of the developed QCT@NG [28]. The dorsal sides of wistar rats were shaved with clippers one day before the test. Rats were divided into two groups: control and treatment (QCT@NG). The test formulation was spread on the shaved area and covered with gauze patch. Any reaction on the skin, such as edema or erythema, was observed on day 1, 7 and 14 post-treatment. Moreover, histopathological examination of the skin was also performed to assess the effects on skin.

##### Acute Toxicity Study

The acute toxicity studies were also performed on male wistar rats (160–200 g) for a period of 14 days [29]. Animals were divided into two groups: control and QCT@NG-treated. In the treatment group, QCT@NG was applied on the skin and observed after the 1st, 7th and 14th days of application. After 14 days of study, animals were anesthesized with diethyl ether and blood was collected from the vein of retro-orbital plexus of the eye. Biochemical parameters, such as Serum Glutamic Pyruvic Transaminase (SGPT), Serum Glutamic Oxaloacetate Transaminase (SGOT), alkaline phosphatase, bilirubin, haemoglobin, total leukocyte count and platelet count were estimated with reported procedures using appropriate diagnostic kits. Further, organs (liver, kidney, heart and spleen) of treated animals were collected and fixed in formalin (10%). Tissues were embedded on paraffin blocks, stained with hematoxylin and eosin dye and were examined under light microscope.

## 3. Results and Discussion

### 3.1. UV-Vis Spectroscopy

The absorption spectrum for QCT showed two absorption bands at 258 and 374 nm, and for calculation purposes the λ_max_ value was considered to be 258 nm. The calibration curve showed a linear relationship between concentration and absorbance (R^2^ = 0.9953) over the tested concentration range, and the equation for linear regression was determined to be y = 0.0233x + 0.0853. 

### 3.2. Solubility Studies

**Figure 1:**
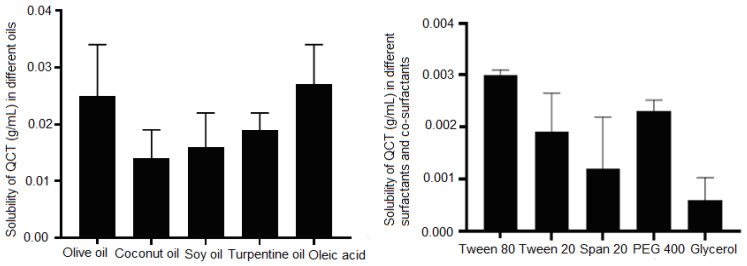
The solubility profile of QCT in different oils, surfactants and co-surfactants.

The solubility of QCT in different oils, surfactants, and cosurfactants was determined and the results are presented in graphical form, as shown in Figure 1. The QCT showed higher solubility in olive oil (25 ± 0.90 mg/mL) and oleic acid (27 ± 0.32 mg/mL) as compared to soy oil (16 ± 0.60 mg/mL), coconut oil (14 ± 0.89 mg/mL) and turpentine oil (19 ± 0.30 mg/mL). It was also observed that Tween 80 and PEG 400 had maximum QCT solubilizing capacities to be used as surfactant and co-surfactant. 

### 3.3. Optimization of QCT@NE Using Design-of-Experiment (DoE)

**Figure 2:**
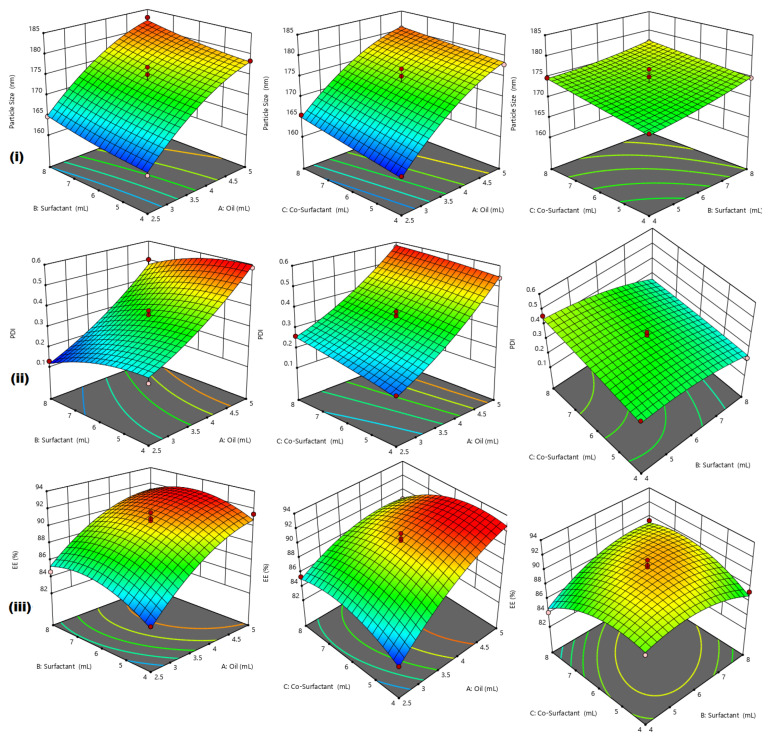
Three-dimensional plots obtained using Box–Behnken design showing effects of factors AB, BC and AC on (**i**) particle size, (**ii**) PDI and (**iii**) %EE.

After collection of data, Analysis of Variance (ANOVA) was used to determine the F-values, *p*-values, and model F-values for the mean globule size (nm) and entrapment efficiency (%EE) values, as responses Y1 (mean globule size) and Y2 (% EE) ranged between 160.6–182.9 nm and 82.74–92.32%, respectively. It was observed that the ratio of maximum to minimum for responses Y1 and Y2 were 1.1388 and 1.1157, respectively. As the values were less than 10, there was no need for transformation. On the basis of sequential model, sums of squares, lack of fit, and model summary statistics, the model necessary for assessing the replies was chosen. Based on the Prob > F value of *p* < 0.0001, low standard deviation, high R2, and reduced projected residual error sum of square (PRESS) value, the quadratic model for both responses was assessed. The proposed model was found to be significant on the basis of the obtained ANOVA data. The three-dimensional (3D) plots obtained after employing the Box–Behnken design are shown in Figure 2.

Signal-to-noise ratio was determined with adequate precision, which is considered to be desirable if it is above 4. Adequate precision for responses Y1 and Y2 was found to be 14.6 and 25.8. The predicted r^2^ of Y1 and Y3 was found to be to be 0.8873 and 0.7709, respectively, indicating adequate signal. Final equations in terms of coded factors for responses Y1 and Y2 were as follows: 

PARTICLE SIZE (Y1): 173.72 + 8.5125 A + 1.8875 B + 1.725 C + 0.1 AB + −0.525 AC + −0.525 BC + −2.585 A^2^ + 0.515 B^2^ + 0.04 C^2^

PDI (Y2): 0.358 + 0.178125 A + −0.062625 B + 0.032 C + 0.0025 AB + −0.01425 AC + −0.02375 BC + 0.0335 A^2^ + −0.0325 B^2^ + −0.00475 C^2^

EE (Y3): 90.62 + 3.46 A + 0.735 B + −0.3375 C + −0.47 AB + −1.56 AC + 1.505 BC + −1.51 A^2^ + −1.58 B^2^ + −1.79 C^2^

Response Y1 consisted of mean globule size and showed positive effects on all three independent variables. It is evident from Figure 2 that the mean globule size increased upon increase of the concentrations of oil, surfactant and co-surfactant. Response Y3 consisted of %EE and the positive coefficients of A and B indicated that the %EE increased with an increase in the concentrations of oil and surfactant, while the negative coefficient of C indicated that the %EE decreased with an increase in the concentration of co-surfactant. Mean globule size plays an important role in the formulation of nanoemulsion during development of emulsions, as it influences the rate and extent of drug release as well as absorption through biological membranes [30].

Perturbation graphs were used to identify the factors that influence responses, and if two factors are present, it helps determine the factor having more influence on the response. A steep slope indicates that the response is sensitive to change in that factor and a flat line indicates no sensitivity. The responses of Y1 showed a steep curvature for factor A and slight bend for factor B and C, which showed that the mean globule size was affected by the concentration of oil. Similarly for the responses of Y3, all the factors showed steep curvatures, which indicated that the entrapment efficiency was affected by the concentrations of oil, surfactant and co-surfactants. All the prepared nanoemulsions were subjected to a thermodynamic stress test and the results indicated that the formulations F2, F3, F5, F7, F9, F10, F16 and F17 showed good thermodynamic stability. Overall comparison of all the prepared QCT@NEs showed that the formulation F10 was most stable, with optimum globule size (173.1 nm) and entrapment efficiency (90.26%). Therefore, F10 formulation was considered to be the best optimized nanoemulsion with oil phase (3.75 mL), surfactant (6 mL) and co-surfactant (6 mL).

### 3.4. Globule Size and PDI

**Figure 3:**
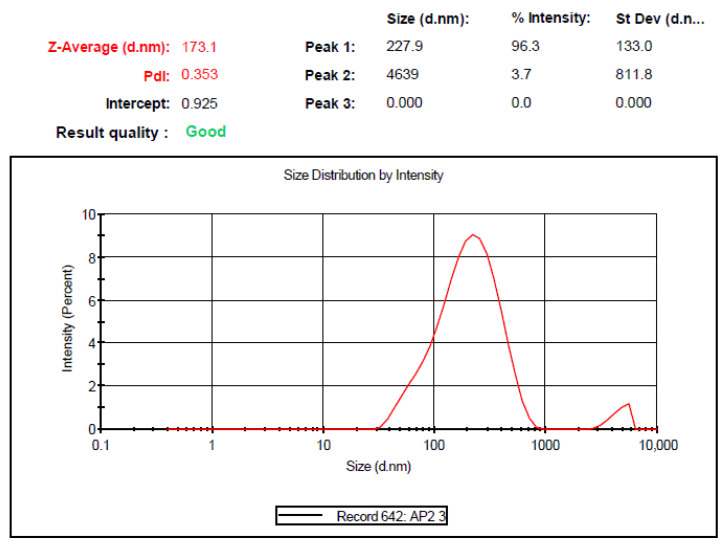
Globule size and PDI of optimized QCT@NE.

The globule size and PDI of the most optimized nanoemulsion was determined using the DLS technique, and the results are shown in Figure 3. The globule size and PDI was found to be 173.1 ± 1.2 nm and 0.353 ± 0.13, respectively. For topical delivery, the particle size needs to be less than 200 nm; therefore, the prepared nanoemulsion was deemed suitable for topical delivery. In comparison to normal emulsion, the smaller particle size of nanoemulsion also results in an increased kinetic stability [31]. It assists in preventing creaming and coalescence, as well as promoting a larger surface area and facilitating the quick transit of nanoemulsion through biological barriers.

### 3.5. Zeta Potential

**Figure 4:**
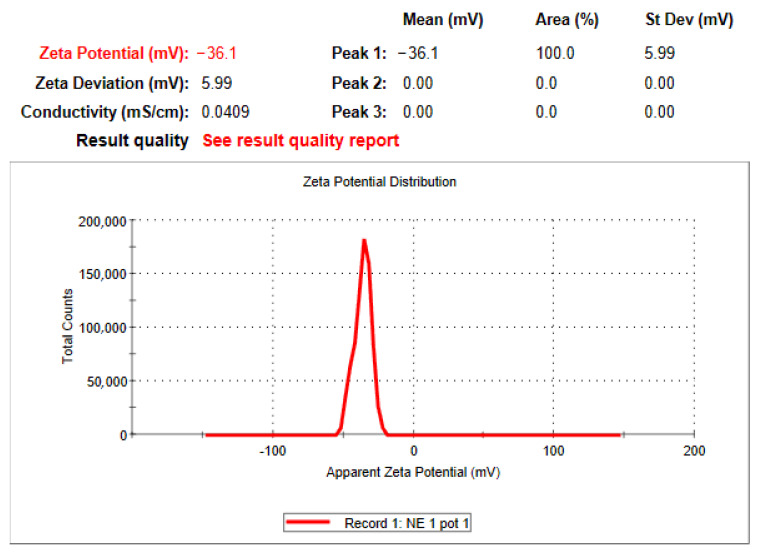
Zeta potential of optimized quercetin-loaded nanoemulsion.

The zeta potential of optimized QCT@NE was also determined using the DLS technique and the results are shown in Figure 4. It showed negative zeta potential value of −36.1 ± 5.99 mV, which might be due to the presence of oils, which help in maintaining the interfacial boundary of the nanoemulsion droplets, leading to a better colloidal system stability. This finding was considered to be satisfactory, as particles with higher zeta potential resist aggregation, while the ones with low zeta potential tend to coagulate [32].

### 3.6. In Vitro Drug Release Study

**Figure 5:**
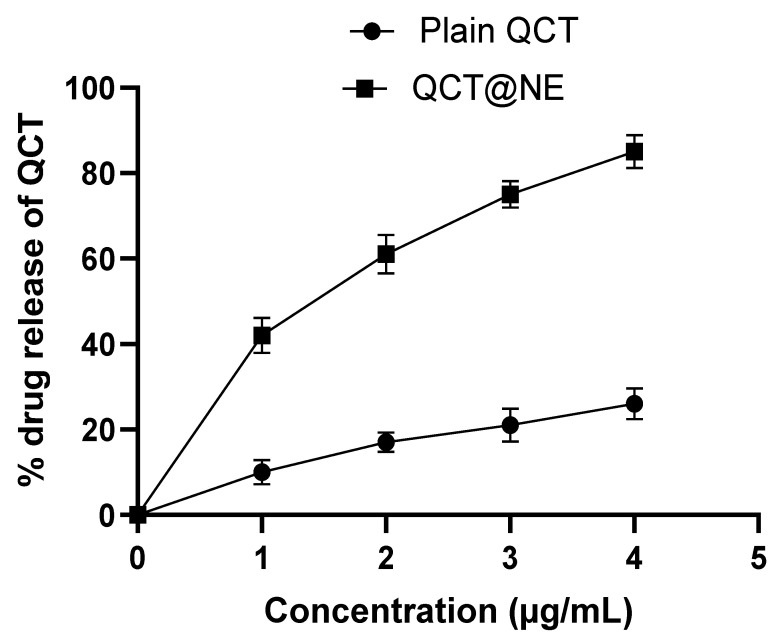
In vitro drug release of QCT from its plain suspension and the optimized nanoemulsion.

The in vitro drug release study was performed using a dialysis membrane, and the data obtained for plain QCT and the most optimized QCT@NE is shown in Figure 5. These findings revealed that 26% of QCT was released from its plain suspension and 85% from the optimized nanoemulsion within 4 h at pH 5. The higher release from the nanoemulsion was due to small globular size, which resulted in large surface area, leading to an increase in the solubility of hydrophobic drugs as well as an increase in the diffusion rate [33]. It was ascertained that the sustained release behaviour of the nanoemulsion would help in the improvement of bioavailability of hydrophobic drugs and to achieve the required therapeutic effect. 

### 3.7. Scanning Electron Microscopy 

**Figure 6:**
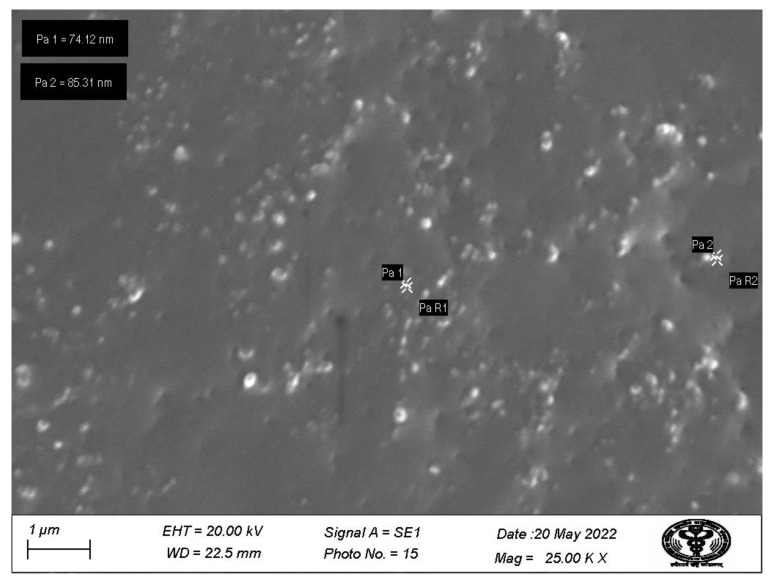
SEM micrograph obtained for QCT@NE.

The optimized nanoemulsion was subjected to SEM to determine the particle size and uniformity, and the result obtained is shown in Figure 6. It was observed that particles were uniformly distributed with very little aggregation. Most of the particles were discrete and were spherical in shape with a size less than 200 nm. 

### 3.8. ATR-FTIR Spectroscopy

The IR spectra of plain QCT, QCT@NE and QCT@NG were obtained using an ATR-FTIR instrument. Plain QCT showed characteristic peaks at 1668 cm^−1^ for C=O stretching, at 3244 cm^−1^ for characteristic absorption band of -OH, and at 1520 and 1558 cm^−1^ corresponding to C=C absorption bands. The FT-IR spectra of QCT@NE showed the same characteristic peaks corresponding to plain QCT with lower intensity, which indicated that the QCT was entrapped in the emulsion system. On the other hand, the FT-IR spectra of QCT@NG showed characteristic peaks at 3500 to 3200 cm^−1^ with higher intensity, indicating the enhanced solubilization of the formulation because of intermolecular hydrogen bonding.

### 3.9. Entrapment Efficiency

The entrapment efficiency of QCT in optimized nanoemulsion was found to be 90.26 ± 0.21%. The higher %EE value was achieved due to the solubility of QCT in olive oil, oleic acid, PEG400 and tween 80 that resulted in its better encapsulation into the formulation.

### 3.10. In Vitro Characterization of QCT@NG

#### 3.10.1. Measurement of pH

The pH of optimized QCT@NG was found to be 5.8, which might be due to the presence of triethanolamine that was added during the preparation of nanoemulgel. The pH of nanoemulgel was acceptable and was expected to be less irritable for human skin as it was closer to the pH of human skin, 5.5. 

#### 3.10.2. Viscosity 

**Figure 7:**
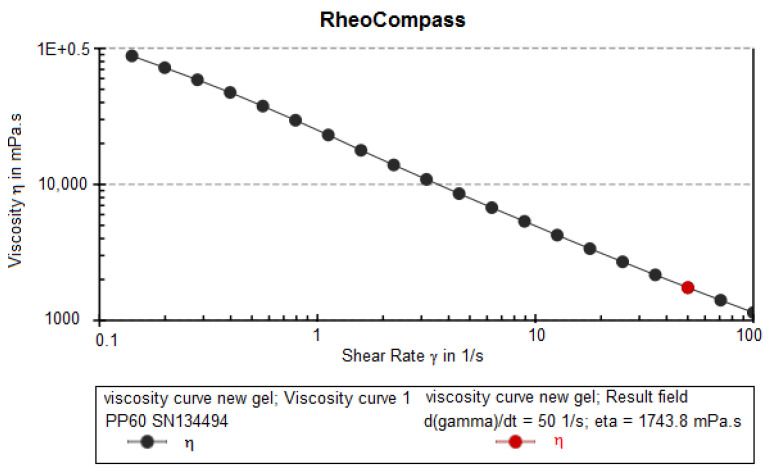
Viscosity vs. shear rate graph of QCT@NG.

Viscosity of the optimized nanoemulgel was calculated to be 100803 ± 1234 cps. It was observed that the viscosity of nanoemulgel decreased upon increasing concentrations of triethanolamine and Carbopol 940 (gelling agent). In addition, a graph (Figure 7) was plotted between viscosity (Y-axis) and shear rate (X-axis), in which viscosity was observed to be decreased linearly by increasing the shear rate and vice versa. It was revealed that the optimized nanoemulgel exhibited non-Newtonian flow, showing thixotrophic behaviour.

#### 3.10.3. Drug Content

Drug content of QCT-loaded nanoemulgel was measured using UV spectroscopy at 258 nm and was found to be 92.3 ± 1.67%.

#### 3.10.4. Texture Profile Analysis

**Figure 8:**
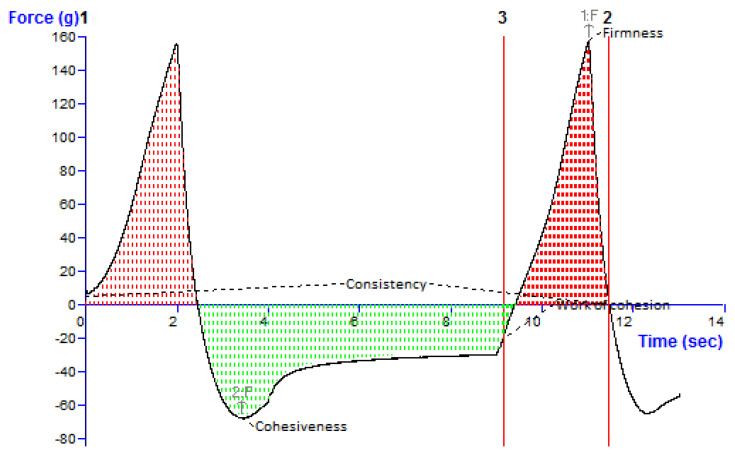
Texture analysis graph of QCT@NG representing the consistency, firmness, cohesiveness and work of cohesion.

The results of texture analysis of the developed QCT@NG are shown in Figure 8. The cohesiveness, consistency, hardness and work of cohesion of optimized nanoemulgel were calculated using the graph and were found to be −68.17 g, 45.42 g/sec, 157.62 g and 140.69 g/sec, respectively. The cohesiveness of the gel had maximum negative force, which indicated that the internal bond strength of the gel was weak and can easily be distorted. The higher degree of gel hardness revealed that an optimum force was required for its application on the skin. The overall texture of the developed nanoemulgel was found to be soft, which was due to the higher work of cohesion [34].

### 3.11. Stability Study

A stability study of QCT@NG was carried out at varied temperatures for a period of two months and the results are summarized in Table 1. Under the two storage conditions, the optimized nanoemulgel was observed to be physically stable, showing no phase separation, caking, and creaming. The percent drug content and pH of nanoemulgel at three time intervals were measured in order to assess degradation, if any. As evident from the table, no considerable change in both pH and drug content were observed, indicating good stability of the developed nanoemulgel. 

### 3.12. Percent Cell Cytotoxicity Study 

**Figure 9:**
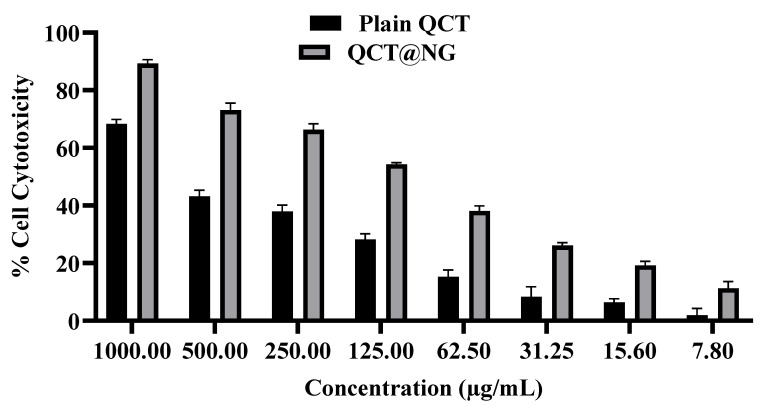
Percent cell cytotoxicity of plain QCT and QCT@NG on A431 human skin cancer cell lines.

The % cell cytotoxicity studies of plain QCT and QCT@NG were performed in vitro on the human skin cancer cell lines A431 and the results are depicted in Figure 9. The findings revealed that the QCT@NG showed significantly increased cytotoxicity on A431 cancer cell lines as compared to plain QCT. This could be due to the inclusion of olive oil, which has been shown to have anti-skin-cancer properties [35]. It was also observed that % cytotoxicity was concentration- as well as time-dependent. The half-maximal inhibitory concentration (IC_50_) of plain QCT and QCT@NG was calculated to be 579.05 and 108.5 µM, respectively, showing excellent potential of QCT@NG for the treatment of skin cancer as compared to plain QCT.

### 3.13. Skin Irritation Study

**Figure 10:**
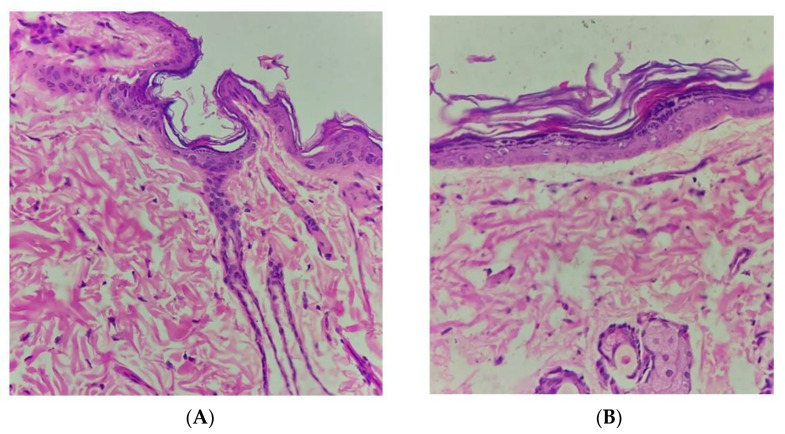
Transverse section of skin of wistar rats following skin irritation study as obtained for the (**A**) control and (**B**) QCT@NG-treated groups.

A skin irritation study on wistar rats was performed for 14 days, and the histopathology of rat skin for the control as well as the QCT@NG-treated group are shown in Figure 10. This study demonstrated that QCT@NG did not trigger any sign of skin irritation due to the presence of herbal ingredients. No signs of edema or erythema were observed in the treatment group as compared to the control. The transverse section of skin of the nanoemulgel-treated group showed tissue lined by keratinized stratified squamous epithelium with normal thickness of epidermis. Dermis was comprised of fibroconnective tissue with interspersed skin adnexal structures, along with hair follicles and sebaceous glands. These parameters were found to be normal and showed no significant differences with the control group. It indicated that the QCT@NG was well-tolerated and did not sensitize the skin. The findings revealed that the developed nanoemulgel did not show any kind of toxicity and was found to be safe for topical application.

### 3.14. Acute Toxicity Study

**Figure 11:**
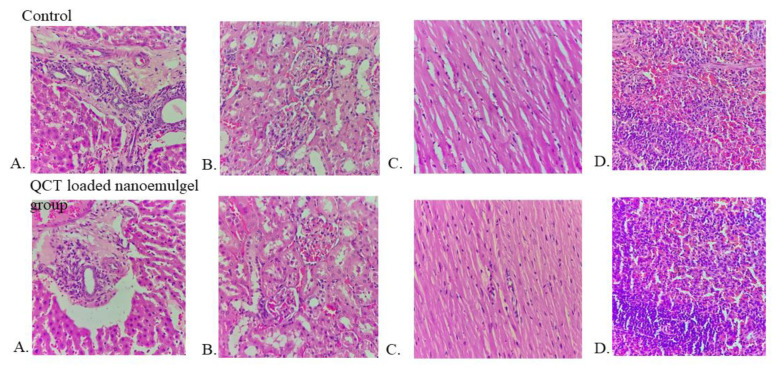
Histopathology study of QCT@NG. (**A**) Liver, (**B**) kidney, (**C**) heart and (**D**) spleen showed no significant changes in the treated group as compared to control.

Hematological analysis after 14 days of treatment with QCT@NG showed no remarkable alterations in any parameter in the treated group as compared to the control. The values of SGPT, SGOT, alkaline phosphatase, bilirubin, hemoglobin, total leukocyte and platelets were found to be 43.3 U/L, 197.1 U/L, and 144.9 U/L, 0.11 mg/dL, 16.98 g/dL, 7.32 × 10^3^/µL and 12.5 × 10^3^/µL, respectively. Figure 11 shows the histopathological results of normal lobular structure, cell plate thickness and polarity of hepatic parenchyma as obtained for the control and QCT@NG-treated groups. The section of heart also shows no necrosis and maintained polarity of myocytes arranged in muscle bundles in both the control and treated groups. The kidney section showed numerous cortex glomeruli with normal capillary loops and mesangial cells. Moreover, proximal convoluted tubule and distal convoluted tubule were observed to be normal in both the control and treated groups. White pulp showed normal-sized primary lymphoid follicle formation and red pulp showed normal cell expansion in spleen section. It was concluded that there were no significant differences observed between QCT@NG-treated wistar rats and the control rats group.

## 4. Conclusions

In conclusion, the QCT@NG was successfully optimized, prepared and assessed for their anticancer potential against human skin cancer cell lines. The prepared nanoemulgel showed ideal characteristics for effective topical delivery of QCT with enhanced skin permeation and effective skin retention, which would render a promising alternative for oral administration of QCT. The topical administration of QCT@NG was safe, as no skin irritation or organ toxicity was observed. Interestingly, the anticancer potential of QCT was observed to be enhanced substantially as compared to the plain QCT. The addition of a gel base to the formulation modifies the emulsion into a non-greasy, viscous and more stable formulation with better patient compliance. The nanoemulgel system offered many propitious and practical features for the topical delivery of QCT and could be an attractive approach compared to other conventional methods. 

## Figures and Tables

**Table 1:** Stability data of the optimized QCT@NG (mean ± S.D.).

Time (Months)	pH	Drug Content (%)
0	5.8 ± 0.32	92.3 ± 1.67
1	5.7 ± 0.45	92.1 ± 1.21
2	5.9 ± 0.11	91.5 ± 2.33

## Data Availability

Not applicable.
